# Analysis of a *lin-42*/*period* Null Allele Implicates All Three Isoforms in Regulation of *Caenorhabditis elegans* Molting and Developmental Timing

**DOI:** 10.1534/g3.116.034165

**Published:** 2016-10-10

**Authors:** Theresa L. B. Edelman, Katherine A. McCulloch, Angela Barr, Christian Frøkjær-Jensen, Erik M. Jorgensen, Ann E. Rougvie

**Affiliations:** * Department of Genetics, Cell Biology, and Development, University of Minnesota, Minneapolis, Minnesota 55454; † Department of Biology, Howard Hughes Medical Institute, University of Utah, Salt Lake City, Utah 84112

**Keywords:** *Caenorhabditis elegans*, *lin-42*, heterochrony, molting

## Abstract

The *Caenorhabditis elegans* heterochronic gene pathway regulates the relative timing of events during postembryonic development. *lin-42*, the worm homolog of the circadian clock gene, *period*, is a critical element of this pathway. *lin-42* function has been defined by a set of hypomorphic alleles that cause precocious phenotypes, in which later developmental events, such as the terminal differentiation of hypodermal cells, occur too early. A subset of alleles also reveals a significant role for *lin-42* in molting; larval stages are lengthened and ecdysis often fails in these mutant animals. *lin-42* is a complex locus, encoding overlapping and nonoverlapping isoforms. Although existing alleles that affect subsets of isoforms have illuminated important and distinct roles for this gene in developmental timing, molting, and the decision to enter the alternative dauer state, it is essential to have a null allele to understand all of the roles of *lin-42* and its individual isoforms. To remedy this problem and discover the null phenotype, we engineered an allele that deletes the entire *lin-42* protein-coding region. *lin-42* null mutants are homozygously viable, but have more severe phenotypes than observed in previously characterized hypomorphic alleles. We also provide additional evidence for this conclusion by using the null allele as a base for reintroducing different isoforms, showing that each isoform can provide heterochronic and molting pathway activities. Transcript levels of the nonoverlapping isoforms appear to be under coordinate temporal regulation, despite being driven by independent promoters. The *lin-42* null allele will continue to be an important tool for dissecting the functions of *lin-42* in molting and developmental timing.

*Caenorhabditis elegans* is a powerful system for studies of developmental time control because of the invariance and precise temporal orchestration with which its cell division patterns are programmed as development proceeds (Sulston and Horvitz 1977; Kimble and Hirsh 1979; Sulston 1983). *C. elegans* develops through embryogenesis and four larval stages, each with a characteristic set of cell divisions and morphogenetic events, prior to becoming reproductively competent adults. Genes that provide temporal cues necessary to specify the appropriate sequence and timing of these postembryonic cell divisions have been identified and termed heterochronic genes (Rougvie and Moss 2013). When heterochronic genes are mutated, specific larval programs are skipped or reiterated, causing subsequent events to occur too early or to be delayed, respectively. For example, mutations in the heterochronic gene *lin-42* cause a precocious phenotype, as demonstrated by terminal differentiation of hypodermal cells occurring one stage too early, during the L3 rather than L4 stage (Abrahante *et al.* 1998; Jeon *et al.* 1999; Tennessen *et al.* 2006). In wild-type animals, *lin-42* temporally restricts this differentiation event, at least in part, by acting as a negative transcriptional regulator of certain miRNA genes, including *let-7* family miRNAs that have prominent roles in the heterochronic gene pathway (Reinhart *et al.* 2000; Abbott *et al.* 2005; Li *et al.* 2005; McCulloch and Rougvie 2014; Perales *et al.* 2014; Van Wynsberghe *et al.* 2014).

*lin-42* functions are broader than control of temporal cell fate in the hypodermis. One additional role is in mediating responses to environmental cues. *lin-42* mutants are hypersensitive to entry into the dauer larva stage, a reversible diapause state that affords survival when growth conditions deteriorate, demonstrating that a wild-type function of *lin-42* is to inhibit dauer formation (Tennessen *et al.* 2010). Another *lin-42* function is in the molting pathway: certain *lin-42* alleles cause variable delays in the molting cycle and a failure of ecdysis, leading to the proposal that *lin-42* may coordinate these activities with hypodermal development (Monsalve *et al.* 2011).

*lin-42* is a particularly intriguing member of the heterochronic gene pathway because it encodes the worm homolog of PERIOD (Jeon *et al.* 1999; Tennessen *et al.* 2006), a core component of the circadian clock in flies and vertebrates, thereby providing a link to another biological timing mechanism. LIN-42 and PERIOD share several regions of homology, including the hallmark PAS protein interaction domains, and smaller SYQ and LT domains that have been shown to interact with circadian clock proteins, including CLOCK and Casein Kinase Iε, in flies and mammals (Chang and Reppert 2003; Lee *et al.* 2004; Sun *et al.* 2010). In flies, the circadian clock is regulated by a transcriptional negative feedback loop between PERIOD/TIMELESS and CLOCK/CYCLE. Activity of a PERIOD/TIMELESS complex mediates repression of CLOCK/CYCLE transcriptional activity, to drive cyclical gene expression.

Interestingly, while PERIOD contains the PAS and SYQ/LT domains in a single protein, the *lin-42* locus encodes multiple protein isoforms, including nonoverlapping proteins that separate these domains. LIN-42B is the longest protein, containing maximal homology to PERIOD proteins as it contains all of the conserved domains (Figure 1A). In contrast, LIN-42A and LIN-42C are nonoverlapping and expressed from distinct promoters (Tennessen *et al.* 2010). LIN-42A contains the SYQ and LT domains, while LIN-42C contains the PAS domains. These two nonoverlapping isoforms provide the opportunity to investigate the function of the PAS and SYQ/LT domains separately. RNA-seq data compiled on WormBase provides strong support for expression of *lin-42a* and *lin-42b*, while support for *lin-42c* comes from 3'RSTs (RACE [Rapid Amplification of cDNA Ends] Sequence Tags) (http://www.wormbase.org, release WS252, date 07-Mar-2016; Salehi-Ashtiani *et al.* 2009). Moreover, a *lin-42* allele that deletes the PAS domain can be rescued by a genomic fragment encompassing the *lin-42c* transcription unit, indicating that *lin-42c* expression can provide function, but this fragment fails to efficiently rescue a premature stop in the LT domain (Tennessen *et al.* 2006). In contrast, *lin-42a* expression can rescue mutations that disrupt either *lin-42a* or *lin-42c*, such as *n1089*, *ve11*, and *ok2385* (Tennessen *et al.* 2006; Monsalve *et al.* 2011) (Figure 1A). These results suggest that the SYQ and LT domain containing isoform is key to LIN-42 function, whereas the PAS-containing isoforms may play a more regulatory role. Supporting this argument is the observation that *n1089*, the PAS domain deletion, and *mg152*, a premature stop codon predicted to eliminate expression of most of the PAS domain, leave Lin-42A intact yet cause a heterochronic phenotype; Lin-42A only rescues these alleles when presumably overexpressed from a multicopy array.

**Figure 1:**
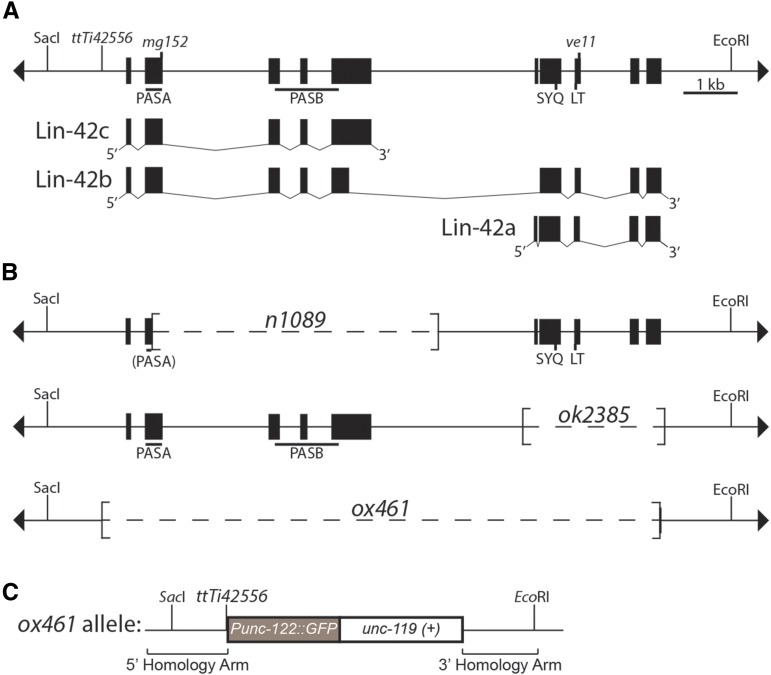
*lin-42(ox461)* deletes the *lin-42* coding region. (A) *lin-42* genomic locus and transcription units. The line with terminal arrowheads represents genomic DNA of the *lin-42* locus drawn with 5′ to the left, which is inverted from WormBase. *lin-42* alleles and the *Mos1* insertion (*ttTi42556*) are indicated above the line. *Sac*I and *Eco*RI sites are included for reference with (B) and (C), but not all recognition sites for these enzymes are shown. The *lin-42* locus produces three transcription units diagrammed below, with filled boxes representing exons: *lin-42a*, *lin-42b*, and *lin-42c*. *lin-42a* and *lin-42c* are nonoverlapping and expressed from distinct promoters (Tennessen *et al.* 2006). Note that the *lin-42* nomenclature used here conforms to that adopted by WormBase and differs from that of pre-2014 publications from the Rougvie laboratory (*e.g.*, Tennessen *et al.* 2006, 2010). (B) *lin-42* deletion alleles. The extent of each deletion is noted in brackets. The *lin-42(n1089)* PASA domain is in parentheses since the majority of the domain is deleted. (C) The *lin-42(ox461)* allele deletes the *lin-42* coding region and replaces it with P*unc-122:gfp* and *C. briggsae unc-119*(+). The fragments used as repair templates in creation of the deletion allele are indicated. See *Materials and Methods* for details.

A tool long missing from the *lin-42* arsenal is a null allele. A deletion allele that eliminates all three isoforms is needed to reveal the *lin-42* null phenotype and allow functional dissection of individual isoform contributions. To remedy this problem and enable further probing of the *lin-42* mechanisms of action, we generated and characterized a *lin-42* null allele using MosDel technology (Frøkjær-Jensen *et al.* 2010) to delete the entire *lin-42* coding region. *lin-42*(*null*) mutants are viable, but have more severe molting and developmental timing defects than previously characterized mutants in which one isoform is left intact.

## Materials and Methods

### Strains and nematode maintenance

*C. elegans* were grown and maintained at 20° on NGM plates seeded with *Escherichia coli* OP50 as previously described (Brenner 1974). Full genotypes of strains used in this work are listed in Table 1.

**Table 1:** Strains used in this study

Strain	Genotype*^a^*	Reference
ARF224	*lin-42(ok2385)*	Monsalve *et al.* (2011)
EG15910	*lin-42(ox460 [*P*unc-122*::*gfp + Cbr-unc-119(+)])*; *unc-119(ed3)*	This work
EG15911	*lin-42(ox461 [*P*unc-122*::*gfp + Cbr-unc-119(+)])*; *unc-119(ed3)*	This work
GR1395	*mgIs49* [P*mlt-10*::*gfp-pest +* P*ttx-3*::*gfp*]	Hayes *et al.* (2006)
IE42556	*ttTi42556*	Vallin *et al.* (2012)
JR667	*unc-119(e2498*::*Tc1)*; *wIs51* [P*scm*::*gfp* + *unc-119(+)*]	Antebi *et al.* (2000)
MT2257	*lin-42(n1089)*	Abrahante *et al.* (1998)
N2	Wild type var. Bristol	Brenner (1974)
RG1514	*ttTi42556*; *unc-119(ed3)*	This work
RG1580	*lin-42(ox461)*; *veEx323* [pHG83 *(lin-42a(gDNA)) + str-1*::*gfp*]	This work; Tennessen *et al.* (2006)
RG1590	*lin-42(ox461)*	This work
RG1650	*lin-42(ox461)*; *wIs51*	This work
RG1665	*lin-42(ox461)*; *veEx593* [pHG83 *+ sur-5*::*gfp*]	This work
RG1739	*lin-42(ox461)*; *veEx651* [pCP2 (P*lin-42b/c*::*lin-42b(cDNA)*::*gfp*::*unc-54 3’UTR*) *+ str-1*::*gfp*]	This work
RG1757	*lin-42(ox461)*; *veEx652* [pCP2 *+ str-1*::*gfp*]	This work
RG1758	*lin-42(ox461)*; *veEx655* [pCP2 *+ str-1*::*gfp*]	This work
RG1786	*lin-42(ox461)*; *veEx594* [pHG83 *+ sur-5*::*gfp*]	This work
RG1791	*lin-42(ox461)*; *gaIs233*	This work
RG1792	*lin-42(ox461)*; *mgIs49*	This work
RG1816	*lin-42(ox461)*; *veEx321* [pMJ13 (P*lin-42b/c*::*lin-42c(gDNA)*::*gfp*::*lin-42 3’UTR*) *+ str-1*::*gfp*]	This work; Tennessen *et al.* (2006)
RG1822	*lin-42(ox461)*; *veEx657* [pMJ13 *+ str-1*::*gfp*]	This work
RG1823	*lin-42(ox461)*; *veEx658* [pMJ13 *+ str-1*::*gfp*]	This work
RG1824	*lin-42(ox461)*; *veEx317* [pMJ13 *+ sur-5*::*gfp*]	This work
RG1825	*lin-42(ox461)*; *veEx319* [pMJ13 *+ sur-5*::*gfp*]	This work; Tennessen *et al.* (2006)
SD1434	*unc-119(ed3)*; *gaIs233* [P*elt-5*::*HIS-24*::*mCherry + unc-119(+)*]	Liu *et al.* (2009)

a Full genotypes are given as appropriate at the first appearance of an allele or transgene array. Key components of plasmids are similarly detailed at the first appearance of the plasmid.

### Generation of a lin-42 null allele

*Mos1*-mediated deletion of the *lin-42* locus was performed essentially as described (Frøkjær-Jensen *et al.* 2010), using a *Mos1* insertion residing 440 bp upstream of the first *lin-42* exon that was obtained from the nemaGENETAG consortium (ttTi42556; Vallin *et al.* 2012) (Figure 1A). The deletion template contained an ∼1.5 kb homology fragment 5′ to the *Mos1* insertion site, and an ∼1.4 kb 3′ homology fragment from the *lin-42a*/b 3′UTR (Figure 1B). The fragments were amplified from wild-type genomic DNA using the following primers that also contain attB sites for Gateway Cloning: 5′ homology fragment, AB25 5′-ggggacagcagctttcttgtacaaagtggaacctaaaactcctcggt-3′/AB26 5′-ggggacaactttgtataataaagttgacgaatcatgttccctgt-3′; 3′ homology fragment, AB27 5′-ggggacaactttgtatagaaaagttggactgaaaattggtgtatgaaca-3′/AB28 5′-ggggactgcttttttgtacaaacttgccgtcttcccgaaaactt-3′. The *lin-42* locus flanking fragments were assembled into pCFJ66, flanking P*unc-122*::*gfp* and *C. briggsae unc-119*, to yield pAB8.

To generate transgenic animals, a mixture of pALB8 (50 ng/µl), pJL43.1 (P*glh-2*::*transposase*, 50 ng/µl), pGH8 (P*rab-3*::*mCherry*, 10 ng/µl), P*myo-2*::*tdTomato* (2.5 ng/µl), and pCFJ104 (P*myo-3*::*mCherry*, 5 ng/µl) was injected into RG1514 *ttTi42556*; *unc-119*(*ed3*). Broods of animals containing extrachromosomal arrays of these plasmids were screened for *unc-119*(+) animals that had lost the extrachromosomal array and thus lacked red fluorescence. These candidate deletion animals were allowed to reproduce and were then genotyped by PCR. DNA sequencing confirmed the appropriate junction fragments using the following primer sets: 5′ junction: AB45 5′-gtaccctcaagggtcctcct-3′/AB46 5′-cccagactttgcatcgaaat-3′; 3′ junction: AB35 5′-cgaaaatttcaaaaagctcgt-3′/AB37 5′-caattcatcccggtttctgt-3′. In each set, one primer lies within the P*unc-122*::*gfp*/*C. briggsae unc-119*(*+*) insertion, while the other primer resides in the genome outside of the homology arms. Two independent *lin-42*(*0*) strains were identified, EG15911 *lin-42*(*ox461*); *unc-119*(*ed3*) and EG15910 *lin-42*(*ox460*); *unc-119*(*ed3*). Each strain was outcrossed three times to generate RG1590 *lin-42*(*ox461*) and RG1591 *lin-42*(*ox460*), respectively.

### Generation of a *lin-42b* minigene and transgenic animals

pCP2 is a *lin-42b* minigene made by subcloning a 5.5 kb *lin-42b/c* promoter fragment from pHG82 (Tennessen *et al.* 2010) onto a *lin-42b* cDNA, with *gfp* coding sequence added just prior to the stop codon, and containing the *unc-54* 3′UTR. pCP2 [P*lin-42b/c*::*lin-42b*::*gfp*::*unc-54*] (5 ng/µl) and *str-1*::*gfp* (100 ng/µl) were injected into N2 hermaphrodites to generate transgenic lines bearing extrachromosomal arrays, which were crossed into *lin-42*(*ox461*) for analysis. The *lin-42c* expressing arrays were made similarly by injecting genomic clone pMJ13 (Jeon *et al.* 1999) at 5 ng/µl together with *str-1*::*gfp* (100 ng/µl) or *sur-5*::*gfp* (75 ng/µl) as a transformation marker.

### Phenotypic analysis

*lin-42*(*ox461*) animals are egg-laying defective, causing adult hermaphrodites to die following the internal hatching of eggs (the so-called “bag-of-worms” phenotype). Because *lin-42* mutant animals are sensitive to growth conditions and their heterochronic phenotypes can be suppressed by starvation (Abrahante *et al.* 1998), eggs were isolated from *lin-42*(*ox461*) animals by hypochlorite treatment, washed in M9 buffer, and plated at low density onto seeded plates so that they would hatch into optimal growth conditions. To monitor postembryonic development starting at the L1 molt, newly hatched larvae were monitored using a Kramer FBS10 microscope until they stopped pumping and entered the first molt, at which time they were singly-picked to seeded 30 mm plates. *lin-42*(*0*) animals were monitored every 2 hr for pumping and ecdysis for 5 d, whereas wild-type control animals were checked hourly until they reached adulthood. Similarly, to quantify the proportion of larval arrest in *lin-42* mutant populations, L1 larvae were singly picked to seeded plates and monitored daily for growth. Animals that failed to reach adulthood by 8 d were classified as arrested. Heterochronic phenotypes were scored at the appropriate developmental stage in animals hatched at low density on seeded plates.

### qPCR analysis

Synchronized populations of worms were generated by hypochlorite treatment of gravid adults to isolate eggs, which were then hatched overnight in M9 buffer. Starved L1s were then plated at a density of 5000–10,000 animals per 10 cm plate. Animals were plated twice, 12 hr apart, to stagger collection times. At each time point, animals were washed off plates with M9, pelleted, and flash frozen in liquid nitrogen. Biological replicates were derived from independent starting populations and performed on different days.

RNA was extracted using TRIzol (Life Technologies) and 425–600 μm glass beads (Sigma) to aid in disrupting the cuticle. Total RNA (5 µg) was treated with DNAse I (Ambion Turbo DNAse kit) to remove any genomic DNA, and then 1 µg was reverse transcribed with 10 U Roche Transcriptor using random primers (0.5 µg; Promega Corp.). qPCR reactions with 12.5 ng of reverse-transcribed sample were run in triplicate, as directed (Roche), on an Eppendorf Realplex Thermocycler using 96-well plates. The following Roche Universal Probe Library (UPL) probe and primers were used for each assay: *lin-42a*, probe #10/TE55 5′-gtacgatcttgcagagccagt-3′/TE56 5′-gaggcttgagtgatggtggt-3′; *lin-42b*, probe #10/TE65 5′-ctttcgaggatgagctgagaa-3′/TE68 5′-ctgatccttgaggcttgagtg; *lin-42c*, probe #146/TE59 5′-aattagacggcgcgagagt-3′/TE60 5′-gccagcatgtgtactttttgc-3′; *mlt-10*, probe #115/TE61 5′-ggcgttgaagaagttcaagag-3′/TE62 5′-cggaacttttcggcttcag-3′; *ama-1*, probe #165/TE72 5′-ggatggaatgtgggttgaga-3′/TE73 5′-gttgtcggtgaggtccattc. Data were collected and analyzed using Realplex 2.0 software. Each time point was normalized to an *ama-1* internal control, and each plate was normalized to the 0 or 6 hr sample within each time-course, as indicated. Data were analyzed using the ΔΔCt method in Microsoft Excel (Livak and Schmittgen 2001). Reactions performed on samples where the reverse transcription step was omitted failed to result in detectable signals (data not shown).

### Data availability

Strains and plasmids used in this work are available upon request. The authors state that all data necessary for confirming the conclusions presented in the article are represented fully within the article.

## Results and Discussion

### Generation of a lin-42 null allele

Genetic analysis of *lin-42* has been complicated by the presence of nonoverlapping isoforms and the lack of a null allele (Tennessen *et al.* 2006). To remedy this situation, the *Mos1*-mediated transposon system (MosDel; (Frøkjær-Jensen *et al.* 2010)) was used to delete the *lin-42* coding region. The *Mos1* insertion ttTi42556 (Vallin *et al.* 2012), 440 bp upstream from the ATG start site (Figure 1A), was targeted by the *Mos1* transposase to generate a double-strand break that was then repaired from a template plasmid containing homology arms outside the *lin-42* coding region (Figure 1B). Two *lin-42*(*null*) alleles were isolated and confirmed by PCR and sequencing: *lin-42*(*ox460*) and *lin-42*(*ox461*). The two deletions are identical, removing 10,226 bp of genomic DNA spanning the *lin-42* coding region and replacing it with *C. briggsae unc-119*(*+*) and a P*unc-122*::*gfp* transgene (Figure 1B). Following three outcrosses to wild type, the strains appeared indistinguishable. *lin-42*(*ox461*) was chosen for detailed analysis and is hereafter referred to as *lin-42*(*0*) for simplicity.

### lin-42(0) is homozygously viable but causes highly penetrant molting defects

*lin-42* is expressed in late stage embryos (Jeon *et al.* 1999), raising the possibility that a null allele could cause embryonic lethality. However, *lin-42*(*0*) animals can be maintained in a homozygous state, and the vast majority of embryos hatch (98%; *n* = 52), indicating that *lin-42* is not essential during embryogenesis.

To further characterize the *lin-42*(*0*) phenotype, we compared it to the phenotypes of two representative hypomorphic alleles, *lin-42*(*n1089*) and *lin-42*(*ok2385*), which each delete one of the two nonoverlapping transcription units. *lin-42*(*n1089*) deletes the majority of *lin-42c*, including the PAS domain and the corresponding portion of *lin-42b*, but leaves *lin-42a* intact (Tennessen *et al.* 2006). Conversely, *lin-42*(*ok2385*) removes *lin-42a* and the SYQ and LT domains, while leaving *lin-42c* intact and truncating *lin-42b* (Monsalve *et al.* 2011). Of these two alleles, *lin-42*(*n1089*) causes the mildest phenotype, largely restricted to heterochronic defects in the hypodermis, while *lin-42*(*ok2385*) is notably more severe, with animals also exhibiting larval arrest and molting defects (Abrahante *et al.* 1998; Monsalve *et al.* 2011).

**Figure 2:**
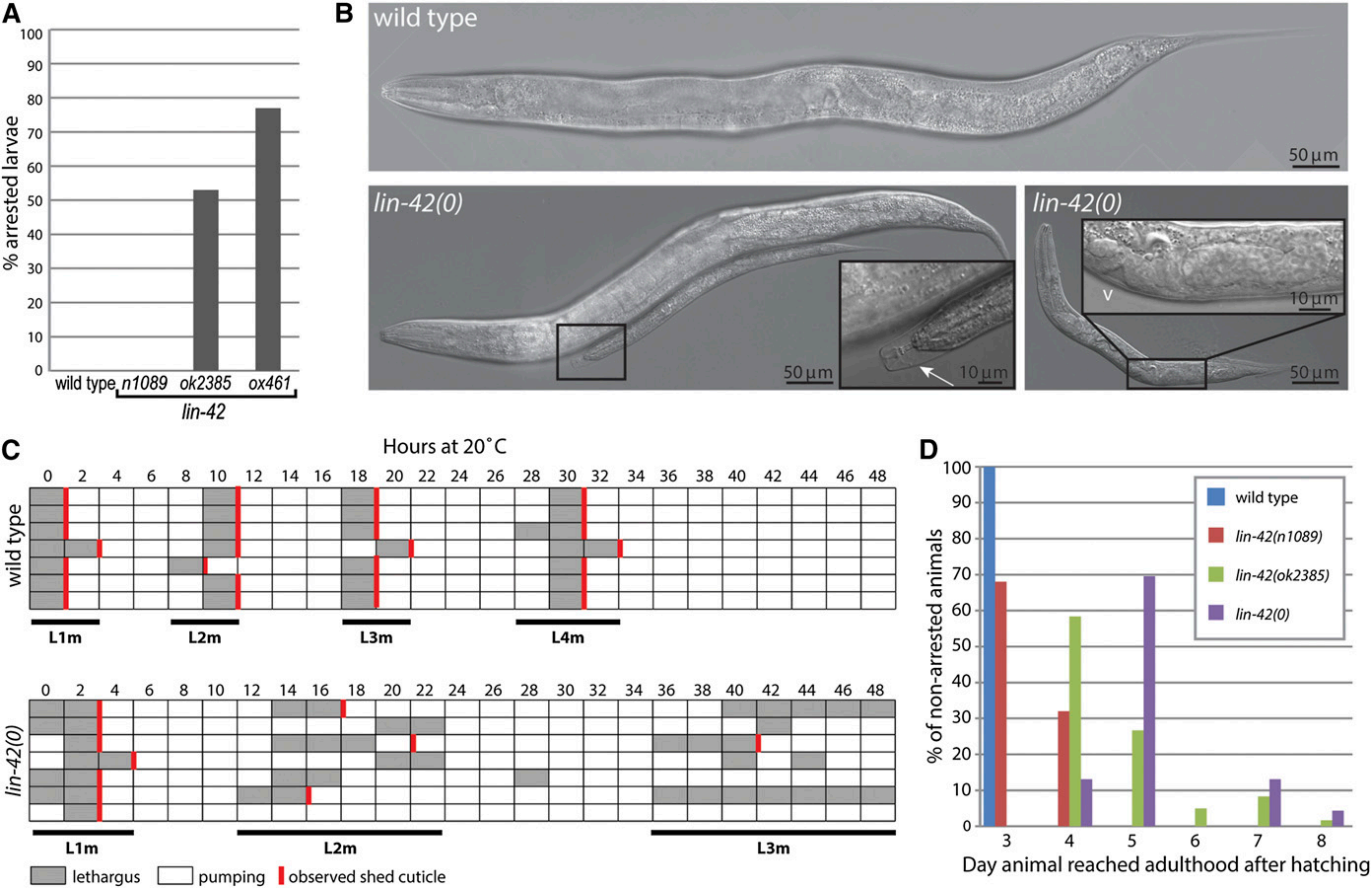
*lin-42(0)* animals exhibit highly penetrant larval arrest and developmental delay phenotypes. (A) *lin-42(0)* mutants have a severe larval arrest phenotype. Individually plated wild-type, *lin-42(n1089)*, *lin-42(ok2385)*, and *lin-42(ox461)* mutants were monitored for developmental progression. The percentage of animals that arrested as larvae and failed to attain adulthood by 8 d posthatching is shown. *n* ≥ 100 for each genotype. (B) Micrographs of wild-type and *lin-42(ox461)* animals 72 hr posthatching. At this time point, all wild-type animals had reached adulthood and were laying eggs, whereas <2% of *lin-42(0)* mutants were adults, and they did not yet contain fertilized eggs, only oocytes. Most *lin-42(0)* animals appeared unable to complete the second larval molt. The left *lin-42(0)* panel contains two animals that are the same chronological age, with an inset showing the smaller animal is trapped in an unshed cuticle (white arrow). The right panel shows a 96 hr animal that appears arrested by size, but has nevertheless begun vulval morphogenesis, marked by the white v in inset. (C) Timing of molts in seven representative wild-type animals (top) and *lin-42(0*) mutants (bottom). Each horizontal row represents an individual animal that was monitored for pharyngeal pumping, lethargus, and ecdysis. Gray shading denotes animals in lethargus and vertical red lines indicate that a shed cuticle was observed on the plate. A total of 68 animals were followed for *lin-42(ox461)* and 10 for wild type. (D) The time of adult onset is delayed in *lin-42* mutants. Animals from (A) that bypassed larval arrest were scored for the day posthatching that they reached adulthood, and graphed as percentage of animals that escaped arrest. *n* = 20, 125, 60, and 23 for wild type, *n1089*, *ok2385*, and *ox461*, respectively.

*lin-42*(*0*) mutants exhibit larval arrest, developmental delays, and molting defects. When the development of individual animals was tracked, 75% of *lin-42*(*0*) animals failed to reach adulthood; they arrested at an early larval stage (Figure 2A). This phenotype is similar to that of *lin-42*(*ok2385*) animals but has a statistically higher penetrance, indicating the upstream region contributes some function (*P* = 0.003, Fisher’s exact test). Strikingly, the larval arrest phenotype is observed in *lin-42*(*0*) and *lin-42*(*ok2385*), but not *lin-42*(*n1089*) mutants (Figure 2A) as previously noted (Monsalve *et al.* 2011), indicating that the PAS domain is dispensable for larval stage progression when *lin-42a* is intact. Arrested animals were often seen trapped in the previous stage cuticle, indicating an inability to ecdyse (Figure 2B), suggesting that molting errors are a causal factor in the larval arrest phenotype.

The *lin-42*(*0*) molting defect appears to result in a growth arrest, perhaps as a consequence of reduced nutrition, rather than a complete developmental arrest in some tissues. Arrested animals exhibited developmental progression based on expression of the motor neuron marker *del-1*::*gfp* (Winnier *et al.* 1999). In wild-type animals, *del-1*::*gfp* expression starts in the VB motor neurons in the early L2, but is repressed in the VA motor neurons, which establish synaptic inputs during the L2 stage. From the late L2 to the adult stage, *del-1*::*gfp* expression is progressively established in the VAs, appearing in an anterior-to-posterior wave. In arrested *lin-42*(*0*) animals, *del-1*::*gfp* expression was initially observed in the 11VB motor neurons, and was progressively activated in the VAs such that by day 5 posthatching, 16 of 20 arrested animals exhibited expression in all VA and VB motor neurons, with the remaining four lacking expression in one or two posterior VAs. In addition, vulval and gonadal development was observed in a small number of arrested animals (Figure 2B, right inset), indicating that these tissues could also developmentally progress. However, the rarity of these animals has so far precluded a thorough analysis of this phenotype.

To better understand the temporal dynamics of postembryonic development in *lin-42*(*0*) mutants, we tracked individual animals beginning at the L1 molt (see *Materials and Methods*). Animals were hatched in the presence of food and synchronized by monitoring entry into L1 lethargus (Figure 2C), a characteristic behavior lasting ∼2 hr at the end of each larval stage, in which the worms cease movement and pharyngeal pumping as a new cuticle is synthesized prior to ecdysis. When compared with wild type, L1 lethargus of *lin-42*(*0*) mutants was lengthened by about 1 hr, but >90% of animals examined completed ecdysis, as evidenced by shed cuticles (Figure 2C and data not shown, *n* = 62). The developmental delays and molting defects became more severe as animals progressed through later larval stages; the population became increasingly more asynchronous and a large percentage of animals failed to shed cuticles. Most *lin-42*(*0*) animals that bypassed early larval arrest executed the third larval molt ∼2.5 d after hatching. The fourth larval stage exhibited the longest delay, with animals requiring at least 24 hr after the L3 molt (L3m) before entering the final period of lethargus (data not shown). These phenotypes mimic those previously described for *lin-42*(*ok2385*) (Monsalve *et al.* 2011).

To compare the relative developmental delay caused by the different *lin-42* alleles, the time required to reach adulthood was assessed for animals that escaped larval arrest. *lin-42*(*0*) mutants had the most severe delay, with a majority of animals requiring 5 d to mature (Figure 2D), while most *lin-42*(*ok2385*) mutants became adults on day 4. A few *lin-42*(*0*) and *lin-42*(*ok2385*) animals required 6–8 d to reach adulthood; these animals were observed to temporarily arrest in an early larval stage before eventually escaping from their cuticle and continuing through development. In contrast, *lin-42*(*n1089*) animals, which have a deletion of the 5′ end of the locus that encodes the PAS domain, exhibit only a mild developmental delay, with ∼70% of animals maturing on day 3, similar to wild-type animals. This analysis reinforces the idea that the SYQ/LT-containing isoforms play a major role in control of molting; however, the enhanced severity of *lin-42*(*0*) relative to the hypomorphic alleles indicates a contribution from the conserved PAS domains encoded by *lin-42b/c* transcripts.

The three *lin-42* alleles examined also form an allelic series with respect to an egg-laying defective (Egl) phenotype: *ox461* > *ok2385* > *n1089* > wild type (Table 2). All *lin-42*(*0*) animals that became fertile adults were Egl, compared with 78% of *lin-42*(*ok2385*) and 18% of *lin-42*(*n1089*) mutants. The proportion of egg-laying competent animals presumably mirrors the relative ability of animals to shed the L4 stage cuticle, thereby allowing eggs to exit through the vulva. A corresponding decrease in average brood size was observed in each strain, likely due to internal hatching of embryos, which eventually leads to death of the hermaphrodite parent, as well as a minor uncharacterized reduction in fertility (Table 2).

**Table 2:** *lin-42* heterochronic and larval arrest phenotypes

	Animals with L3m Alae (%)					Animals That Reached Adulthood		
Genotype	Complete	Partial	None	% Arrest*^a^*	No. Seam Cell*^b^*	Egl (%)	Fertile (%)	Brood Size*^c^*
Wild type	0	0	100	0	nd	0	100	336.7 ± 37.7
*lin-42(n1089)*	21	76	3	0	nd	18	100	69.1 ± 67.5
*lin-42(ok2385)*	13	83	4	53	nd	78	85	36.5 ± 46.1
*lin-42(ox461)*	69	31	0	77	nd	100	72	7.6 ± 5.2
P*elt-5*::*mCherry*	0	0	100	nd	15.7 ± 0.75	nd	nd	nd
*ox461*; P*elt-5*::*mCherry*	59	41	0	nd	16.0 ± 0.56	nd	nd	nd
P*scm*::*gfp*	0	0	100	nd	16.0 ± 0.00	nd	nd	nd
*ox461*; P*scm*::*gfp*	0	5	95	nd	16.0 ± 0.32	nd	nd	nd
P*mlt-10*::*gfp*	0	0	100	nd	nd	nd	nd	nd
*ox461*; P*mlt-10*::*gfp*	9	14	77	nd	nd	nd	nd	nd
*lin-42a* rescue								
* ox461*; *veEx323*	0	14	86	0	nd	3	100	147.0 ± 25.3
* ox461*; *veEx593*	0	28	72	2	nd	1	99	189.3 ± 70.8
* ox461*; *veEx594**^d^*	0	0	100	4	nd	10	100	106.0 ± 57.9
*lin-42b* rescue								
* ox461*; *veEx651*	0	4	96	2	nd	4	100	193.3 ± 71.1
* ox461*; *veEx652*	0	4	96	2	nd	6	100	208.8 ± 84.2
* ox461*; *veEx655*	0	6	94	14	nd	16	100	186.5 ± 66.1
*lin-42c* rescue								
* ox461*; *veEx317*	17	48	35	59	nd	85	85	12.3 ± 11.7
* ox461*; *veEx319*	0	67	33	61	nd	76	86	19.5 ± 30.4
* ox461*; *veEx321*	22	74	4	37	nd	97	81	13.1 ± 25.9
* ox461*; *veEx657*	0	19	81	83	nd	75	89	20.8 ± 32.1
* ox461*; *veEx658*	4	68	28	74	nd	46	97	45.3 ± 49.5

All animals assayed arose from eggs hatched onto seeded plates at low density. *n* ≥ 20 for all analyses except brood size of *ox461* and *ox461*; *veEx657* animals, where *n* = 16.

a Percentage of animals that arrested as young larvae.

b P*elt-5*::*mCherry* strains were scored as young adults, and P*scm*::*gfp* strains were scored during the L3 molt.

c Average number of progeny from fertile adults; sterile animals were not included in the calculation.

d This strain exhibited some embryonic lethality; non-Egl adults laid an average of 33 ± 23 dead eggs.

### lin-42(0) heterochronic defects are more severe than those caused by hypomorphic alleles

**Figure 3:**
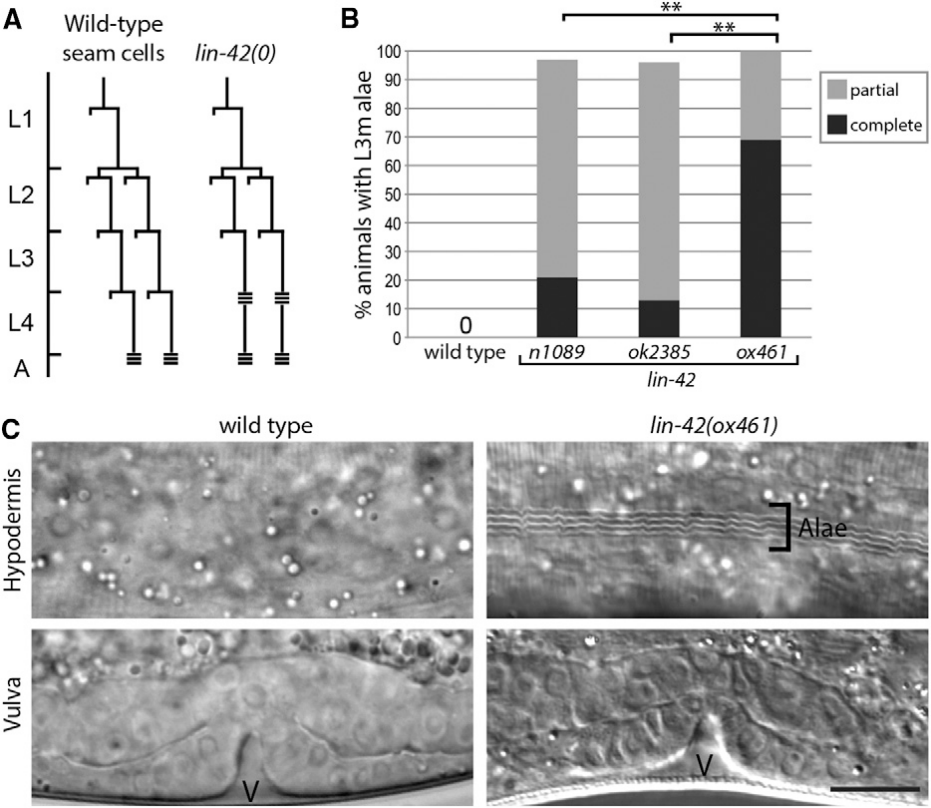
*lin-42(0)* mutants have a precocious heterochronic phenotype. (A) *lin-42* mutations cause precocious seam cell development. Shown is a representative seam cell lineage (V1–V4, V6) diagram in wild-type and *lin-42(0)* mutants. A horizontal line indicates a cell division and triple horizontal bars indicate alae formation. Developmental stages are relative to the molts, and the actual intermolt periods of wild-type and *lin-42(0)* animals are different (see Figure 2C). (B) *lin-42(0)* mutants have a more severe heterochronic defect than *lin-42(lf)* animals. Wild-type, *lin-42(n1089)*, *lin-42(ok2385)*, and *lin-42(ox461)* animals were analyzed at the L3 molt for alae formation. Animals were scored for either complete, partial (alae with gaps), or no alae. *n* ≥ 20 for each strain. **P < 0.0001, Fisher’s exact test. (C) *lin-42(0)* animals form precocious alae at the L3 molt stage. Micrographs of representative wild-type and *lin-42(0)* animals at the third molt are shown, with an image of cuticle in the top row and vulval morphogenesis (v) of the animal below to denote staging. Bar, 10 µm applies to all panels.

*lin-42* was defined by mutations with a precocious heterochronic phenotype in which hypodermal seam cells terminally differentiate and produce a characteristic adult cuticle with alae one stage too early at the L3m (Abrahante *et al.* 1998; Jeon *et al.* 1999) (Figure 3, A and B). In order to score this phenotype in a population of *lin-42*(*ox461*) animals, which develop asynchronously through the larval stages, it was necessary to reliably identify L3 stage animals. Tracking of individual animals revealed that, in *lin-42*(*0*) animals that did not arrest, vulval divisions occurred during the L3 stage as in the wild type, and vulval morphogenesis had proceeded to the invagination stage by the L3m, appearing at least superficially wild type (Figure 3C). Vulval morphology was thus used to identify L3m *lin-42*(*0*) animals from roughly staged populations of animals.

Partial or complete precocious alae was observed in all *lin-42*(*0*) animals at the L3m. This defect is significantly stronger than that of either *lin-42*(*n1089*) or *lin-42*(*ok2385*) mutants (*P* < 0.0001, Fisher’s exact test), with a larger proportion of animals forming complete alae at the L3m (Figure 3, B and C). The observation that *lin-42*(*0*) is more severe than either *lin-42*(*n1089*) or *lin-42*(*ok2385*) suggests the isoform present in each hypomorphic allele contributes some level of function, even though they are distinct proteins.

In addition to defects in the timing of seam cell terminal differentiation, several heterochronic mutants have altered seam cell number due to the skipping or reiteration of the proliferative division normally observed at the beginning of the second larval stage in wild-type animals (Figure 3A), when seam cell number is increased from 10 to 16 on each lateral side (Sulston and Horvitz 1977). Prior results that link *lin-42* activity to control of the proliferative division prompted assessment of seam cell number in *lin-42*(*0*) animals. Notably, *lin-42* negatively regulates expression of *mir-48* (McCulloch and Rougvie 2014; Perales *et al.* 2014; Van Wynsberghe *et al.* 2014), a miRNA that when overexpressed, results in reduced seam cell number (Li *et al.* 2005). In addition, when grown at the permissive temperature of 15°, *lin-14*(*n179ts*); *lin-42*(*n1089*) animals skip the L2 stage proliferative division, a phenotype not observed in either single mutant (Liu 1990). We used the P*elt-5*::*his-24*::*mCherry* (Liu *et al.* 2009) hypodermal marker as an aid in scoring seam cell number. *lin-42*(*0*); P*elt-5*::*his-24*::*mCherry* animals hatch with a wild-type number of seam cells on each lateral side (9.95 ± 0.22) that proliferate appropriately, resulting in adults with the full complement of 16 ± 0.56 (Table 2). Thus, *lin-42* activity is not essential for seam cell specification or proliferation.

P*elt-5*::*his-24*::*mCherry* was used to monitor seam cell number rather than *scm*::*gfp*, a standard tool used in the assessment of seam cell number and morphology in heterochronic mutants (Antebi *et al.* 2000; Lehrbach *et al.* 2009; Hada *et al.* 2010; Chu *et al.* 2014), because the latter reporter strongly suppressed the *lin-42*(*0*) precocious alae phenotype (Table 2). The observed suppression was not specific to the integrated *scm*::*gfp* transgene used (*wIs51* [*scm*::*gfp* + *unc-119*(+)]); similar suppression was also observed in animals with an integrated *mlt-10*::*gfp* reporter (*mgIs49* [P*mlt-10*::*gfp-pest +* P*ttx-3*::*gfp*]) (Hayes *et al.* 2006). One commonality between these transgene reporters is that P*mlt-10*::*gfp* and *scm*::*gfp* are both expressed in the hypodermis, although primarily in distinct cells. P*mlt-10*::*gfp* has a cyclical expression pattern in hyp7, the main body hypodermal syncytium, peaking ∼4 hr prior to ecdysis (Meli *et al.* 2010), whereas *scm*::*gfp* is predominantly expressed in the hypodermal seam cells throughout the larval stages (Terns *et al.* 1997; Mohler *et al.* 1998; Koppen *et al.* 2001). One possibility is that given the multicopy nature of the transgene arrays, the *scm*::*gfp* and P*mlt-10*::*gfp* promoters may titrate out a factor whose precocious expression is required for alae synthesis at the L3m. Although the mechanism of suppression is not clear, caution should be used when scoring heterochronic phenotypes in mutants containing these reporters. Even though P*elt-5*::*his-24*::*mCherry* is expressed in seam and hyp7 cells during larval stages and maintained in adult seam cells, suppression was not observed in animals with this reporter (Table 2).

### Each isoform can provide some lin-42 activity

Previous studies showed that expression of the SYQ/LT domain encoding transcript *lin-42a* from multicopy arrays can rescue both *lin-42*(*ve11*), a premature stop in *lin-42a* (Figure 1A), and the *lin-42*c deletion *lin-42*(*n1089*), whereas expression of *lin-42c* only rescues *lin-42*(*n1089*) efficiently (Tennessen *et al.* 2006). The availability of a null allele afforded us the opportunity to more fully test the ability of each isoform to rescue the heterochronic, larval arrest, and egg-laying phenotypes (Jeon *et al.* 1999; Tennessen *et al.* 2006). Transgenic expression of *lin-42a* strongly rescued both the heterochronic and larval arrest phenotypes of *lin-42*(*0*) animals (Table 2), indicating that this isoform is sufficient to control these processes. However, there is likely to be a contribution from overexpression at play, because the *n1089* and *mg152* alleles result in heterochronic defects, demonstrating that endogenous *lin-42a*, although sufficient for allowing larval progression, is insufficient to regulate the timing of hypodermal fates.

To test the rescuing ability of *lin-42b*, it was first necessary to construct a *lin-42b* minigene by fusing the *lin-42b/c* promoter to a *lin-42b* cDNA with *gfp* inserted prior to the stop codon. Use of a *lin-42b* cDNA omits the 3.6 kb intron between exons 5 and 6, which harbors the *lin-42a* promoter and thus prevents independent expression of *lin-42a* (Figure 1A). P*lin-42b/c*::*lin-42b*(*cDNA*)::*gfp*::*unc-54* 3′UTR expression rescued both the heterochronic and molting defects of *lin-42*(*0*) animals at least as well as *lin-42a* expression (Table 2). Moreover, this result indicates that the essential spatial and temporal enhancers that drive *lin-42b/c* expression are located upstream of the *lin-42* coding region and/or reside in exons.

The ability of *lin-42c* expression to rescue the null was less robust than was observed for either *lin-42b* or *lin-42a* (Table 2). *lin-42c* only weakly rescued the precocious alae phenotype, and four of five lines tested showed little, if any, rescue of the larval arrest phenotype. As in nontransgenic null mutants, the arrested animals were often observed to be trapped in a previous stage cuticle, indicating failure to rescue molting defects. The effects on egg-laying ability, fertility, and brood size were also greatly dampened relative to the rescuing abilities of *lin-42b* or *lin-42a*. We have not ruled out the possibility that the *lin-42b/c* promoter itself contributes to the minimal rescue observed with *lin-42c*. We note that the extent of rescue among *lin-42c* lines appeared somewhat more variable than for *lin-42a* or *lin-42b* rescue, and the variability does not correlate with the coinjection marker used (*str-1*::*gfp vs. sur-5*::*gfp*; see Table 1 and Table 2). Another possibility is that the variable rescue could reflect a requirement for an optimal expression range that may vary for the different phenotypes scored. For example, GFP is readily detected in *ox461*; *veEx658* animals, which show rescue of the alae phenotype but not larval arrest, and conversely, GFP is not detected in *ox461*; *veEx321* animals which show significant rescue of the larval arrest phenotype but less extensive rescue of precocious alae. Regardless of the reason underlying the *lin-42c* results, the rescue experiments clearly demonstrate that the *lin-42c*–expressing construct is dramatically less efficient than either *lin-42a* or *lin-42b* at providing function.

### lin-42 transcripts have similar accumulation patterns

**Figure 4:**
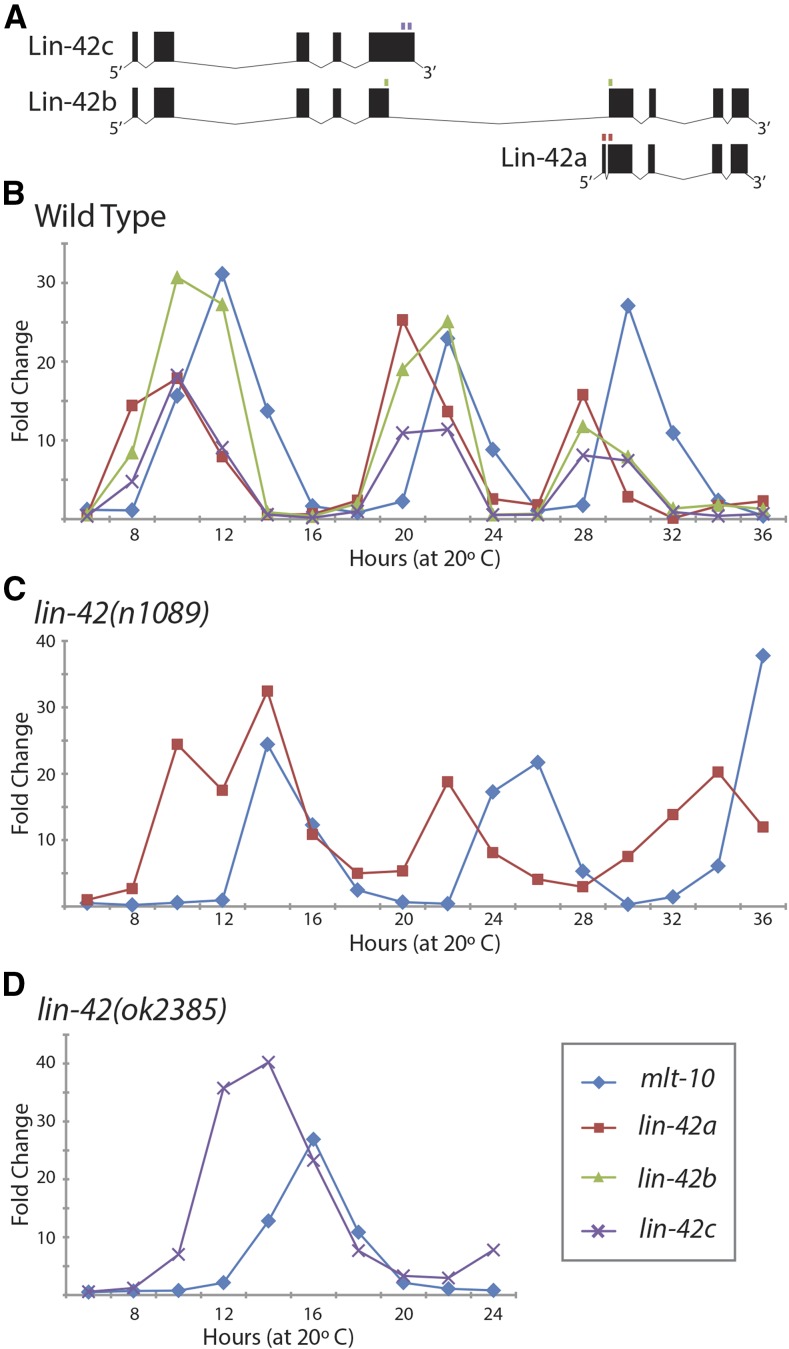
Levels of three *lin-42* transcripts cycle in unison. (A) *lin-42* transcript diagrams with small boxes above indicating the locations of primer sets used in qPCR assays: *lin-42a* (red), *lin-42b* (green), and *lin-42c* (purple). *lin-42a* and *lin-42b* assays are intron spanning and recognize a single transcript, whereas *lin-42c* does not contain a unique intron; its primer could also amplify the *lin-42b* primary transcript. (B–D) Representative time courses of *lin-42a*, *lin-42b*, and *lin-42c* accumulation, relative to the time of *mlt-10* expression, in wild-type and *lin-42* mutants. Two independent biological replicas for each genotype are shown in Figure S1, along with primer controls. (B) Wild type with time points normalized to 0 hr. (C and D) *lin-42a* and *lin-42c* levels cycle in *lin-42* mutant backgrounds. Time points within each assay are normalized to 6 hr. (C) *lin-42a* message levels in *lin-42(n1089)* mutants. (D) *lin-42c* message levels in *lin-42(ok2385)* mutants from 6 to 24 hr.

*lin-42a* is expressed from a different promoter than are *lin-42b* and *lin-42c* (Tennessen *et al.* 2006), raising the question as to whether their temporal expression patterns differ. *lin-42* expression levels were reported to be oscillatory using a PCR approach that would detect both *lin-42b* and *lin-42c* (Jeon *et al.* 1999), with levels peaking in the intermolts and becoming undetectable during the molts, and subsequent reports confirmed a pulsatile expression pattern for *lin-42* using a variety of methods (Gissendanner *et al.* 2004; Hendriks *et al.* 2014). However, assays of promoter fusions to destabilized fluorescent reporter proteins have led to conflicting reports about the relative order of *lin-42a vs. lin-42b* expression (Monsalve *et al.* 2011; Perales *et al.* 2014). As an alternative method to address this question, we measured endogenous levels of each *lin-42* transcript, using the Roche UPL RT-qPCR assay, and compared their temporal expression profiles throughout development. The primer sets were specific, as they failed to detect a product when the corresponding deletion allele was assayed (Supplemental Material, Figure S1). Primers for *lin-42a* and *lin-42b* spanned introns and were confirmed to detect mature transcripts; however, an intron-spanning assay specific to *lin-42c* cannot be designed, and the assay used could also detect an unspliced *lin-42b* transcript (Figure 4A). Therefore, the most informative comparison is between *lin-42a* and *lin-42b* message levels. RNA was prepared from synchronized populations of wild-type animals collected at 2-hr intervals from 6 to 36 hr and transcript levels were assayed, using endogenous *mlt-10* expression as an internal control to mark developmental time and monitor synchrony. *mlt-10* message levels also oscillate, peaking ∼4 hr before each lethargus (Figure 4) (Frand *et al.* 2005; McCulloch and Rougvie 2014). The expression patterns of the *lin-42* transcripts were found to be highly similar to each other (Figure 4), peaking at approximately the same time and returning to a low basal level before each molt (∼4 hr after the *mlt-10* peak), despite being derived from two different promoters.

The availability of transcript-specific reagents and deletion alleles provided the opportunity to test whether the temporal expression patterns of *lin-42a* and *lin-42b/c* are interdependent. In *lin-42*(*n1089*) mutants, *lin-42a* and *mlt-10* transcript levels cycled with each larval stage (Figure 4C), similar to the wild-type pattern, but with a longer period, consistent with the mild developmental delay observed in this strain (Figure 2D). *lin-42*(*ok2385*) animals only maintain reasonable synchrony until the first larval molt, limiting examination of *lin-42* expression in these animals to the L1 stage. *lin-42b/c* undergoes a complete expression cycle during the L1 stage in *lin-42*(*ok2385*) mutants, peaking at 12–14 hr and returning to a low basal level before the molt (Figure 4D). Together, these results indicate that the cyclical expression patterns of *lin-42a* and *lin-42b/c* do not require the presence of the other *lin-42* transcripts.

### Conclusions

A longstanding question in the *C. elegans* developmental timing field has been whether complete lack of *lin-42/period* activity would confer novel heterochronic phenotypes. This question has been unanswered due to lack of alleles that eliminated all isoforms. We remedied this problem by generating a null allele that deletes the entire *lin-42* coding region. Phenotypic analysis has so far revealed no new heterochronic defects and that the *lin-42*(*0*) mutant has similar, but more penetrant, heterochronic and molting defects than the hypomorphic alleles *lin-42*(*n1089*) and *lin-42*(*ok2385*). These results demonstrate that the two nonoverlapping transcription units, *lin-42a* and *lin-42c*, present in *lin-42*(*n1089*) and *lin-42*(*ok2385*) mutants respectively, each confer some level of function.

The three *lin-42* transcription units appear highly similar in their temporal accumulation patterns, peaking at a similar time during each intermolt and becoming undetectable during each molt. *lin-42a* and *lin-42b/c* maintain cyclical expression patterns in *lin-42*(*n1089*) and *lin-42*(*ok2385*) mutants respectively, indicating their dynamic expression patterns do not require the missing isoform(s).

Transgene expression experiments reveal that the most important isoforms are LIN-42A and LIN-42B. LIN-42A lacks PERIOD’s hallmark PAS domain, but contains the smaller conserved SYQ/LT domains, and LIN-42B contains all three. *lin-42a* or *lin-42b* expression strongly rescues *lin-42*(*0*) mutants for all parameters assessed, whereas PAS domain encoding *lin-42c* only mildly rescues *lin-42*(*0*) heterochronic defects. The strong rescuing ability of *lin-42b* indicates that independent expression of the short isoforms is not absolutely required for function.

Aside from the fact that SYQ/LT domain containing isoforms are key, the functions of the conserved motifs in LIN-42 are unclear. Indeed, to date, no missense alleles of *lin-42* have been reported that cause phenotypes; all alleles are deletions or result in premature stop codons. The availability of a null allele will now facilitate structure function analysis, and in conjunction with CRISPR-Cas9 genome engineering approaches, should allow correlation of functions with isoforms, and more precisely, to the domains they harbor and the amino acids that comprise them.

*lin-42* has a well-defined role as a negative transcriptional regulator of miRNA genes, including some that function in the heterochronic gene pathway (McCulloch and Rougvie 2014; Perales *et al.* 2014; Van Wynsberghe *et al.* 2014), potentially allowing *lin-42* to indirectly coordinate translation of many downstream messenger RNAs. Initial chromatin immunoprecipitation coupled to sequencing (ChIP-seq) experiments have indeed found LIN-42 associated with chromatin at miRNA promoters, but in addition, LIN-42 is found near the transcription starts of protein-coding genes (Perales *et al.* 2014). Key goals for the future will be to validate these targets and begin to partition them among *lin-42*’s various roles, which include the molting, timing, and dauer formation pathways, as well as to determine whether the *lin-42* isoforms are differentially recruited to promoters. Interestingly, levels of LIN-42C present in *lin-42*(*n1089*) mutants are sufficient to carry out the molting function, but not the heterochronic function, perhaps reflecting a division of labor among isoforms. One possible model is that the region spanning the SYQ and LT domains is important for LIN-42 association with most targets and, similar to PERIOD, acts to block the action of transcriptional activators. The PAS domain may provide a more regulatory function, enhancing the activity of the SYQ/LT region, or allowing it access a wider array of target genes.

## Supplementary Material

Supplemental Material
