# Supplementary material for: Analysis of a lin-42/period Null Allele Implicates All Three Isoforms in Regulation of Caenorhabditis elegans Molting and Developmental Timing
Source: G3 (Bethesda). 2016 Oct 10;6(12):4077–86. doi: 10.1534/g3.116.034165 (PMC5144976; doi:10.1534/g3.116.034165)
Supplement: Supplemental Material [file supp_6_12_4077__index.html]

Analysis of a lin-42/Period Null Allele Implicates All Three Isoforms in Regulation of Caenorhabditis elegans Molting and Developmental Timing — Analysis of a lin-42/period Null Allele Implicates All Three Isoforms in Regulation of Caenorhabditis elegans Molting and Developmental Timing — Supplemental Material 

# Analysis of a *lin-42*/*period* Null Allele Implicates All Three Isoforms in Regulation of *Caenorhabditis elegans* Molting and Developmental Timing

## Supplemental Material for Edelman *et al*, 2016

**Files in this Data Supplement:**

- Figure S1 - Biological replicates of *lin-42* transcript accumulation patterns.
