# Supplementary material for: Analysis of a lin-42/period Null Allele Implicates All Three Isoforms in Regulation of Caenorhabditis elegans Molting and Developmental Timing
Source: G3 (Bethesda). 2016 Oct 10;6(12):4077–86. doi: 10.1534/g3.116.034165 (PMC5144976; doi:10.1534/g3.116.034165)
Supplement: Supplemental Material [file supp_g3.116.034165_FigureS1.pdf]

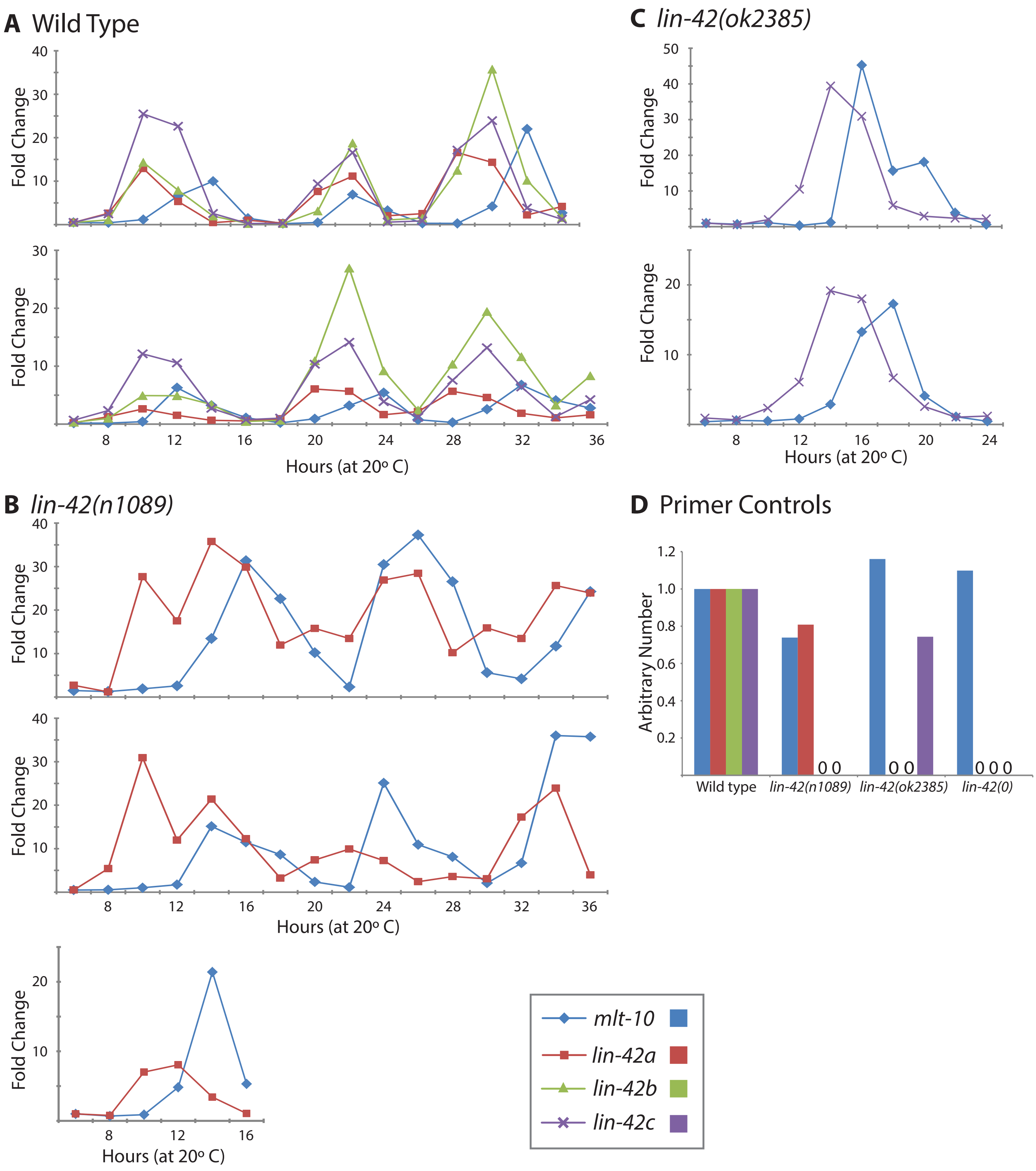

A Wild Type
Fold Change
Hours (at 20º C)
B lin-42(n1089)
Fold Change
Hours (at 20º C)
C lin-42(ok2385)
Fold Change
Hours (at 20º C)
D Primer Controls
Arbitrary Number
Wild type
lin-42(n1089)
lin-42(ok2385)
lin-42(0)
0 0
0 0
0 0 0
mlt-10
lin-42a
lin-42b
lin-42c

Figure S1: Biological replicates of lin-42 transcript accumulation patterns.

(A-C) Expression of *lin-42a*, l*in-42b*, and *lin-42c* was measured relative to *mlt-10* in a total of three biological replicates (one replicate is displayed in Figure 4) for (A) wild type, (B) *lin-42(n1089)*, and (C) *lin-42(ok2385)*. Time points within each assay are normalized to 0 or 6 hr. (B) The decrease in *lin-42a* expression at 12hrs in each *lin-42(n1089)* time course is an artifact of staggering populations, since the decrease is not observed when animals were collected continuously from 6-16 hrs (third panel). (D) *lin-42* gene expression assays are specific to the intended target. Transcript levels were measured in mixed-stage populations of wild type, *lin-42(n1089)*, *lin-42(ok2385)*, and *lin-42(0)* to demonstrate the specificity of each assay. ΔCt values ([target Ct] – [*ama-1* Ct]) are normalized to wild type.
